# Accounting for the health risk of probiotics

**DOI:** 10.1016/j.heliyon.2024.e27908

**Published:** 2024-03-10

**Authors:** Xiangyi Liu, Haiyi Zhao, Aloysius Wong

**Affiliations:** a Department of Biology, College of Science, Mathematics and Technology, Wenzhou-Kean University, 88 Daxue Road, Ouhai, Wenzhou, Zhejiang Province, 325060, China; b Department of Biology, Dorothy and George Hennings College of Science, Mathematics and Technology, Kean, University, 1000 Morris Ave, Union, NJ, 07083, USA; c Wenzhou Municipal Key Lab for Applied Biomedical and Biopharmaceutical Informatics, Ouhai, Wenzhou, Zhejiang Province, 325060, China; d Zhejiang Bioinformatics International Science and Technology Cooperation Center, Ouhai, Wenzhou, Zhejiang Province, 325060, China

**Keywords:** Probiotics, *Lactobacillus*, *Bifidobacterium*, Antibiotic resistance, Antimicrobial resistance genes, Horizontal gene transfer

## Abstract

Probiotics have long been associated with a myriad of health benefits, so much so that their adverse effects whether mild or severe, are often neglected or overshadowed by the enormous volume of articles describing their beneficial effects in the current literature. Recent evidence has demonstrated several health risks of probiotics that warrant serious reconsideration of their applications and further investigations. This review aims to highlight studies that report on how probiotics might cause opportunistic systemic and local infections, detrimental immunological effects, metabolic disturbance, allergic reactions, and facilitating the spread of antimicrobial resistance. To offer a recent account of the literature, articles within the last five years were prioritized. The narration of these evidence was based on the nature of the studies in the following order of preference: clinical studies or human samples, in vivo or animal models, in situ, in vitro and/or in silico. We hope that this review will inform consumers, food scientists, and medical practitioners, on the health risks, while also encouraging research that will focus on and clarify the adverse effects of probiotics.

## Highlights

• The adverse effects of probiotics currently lack both scientific and public attention.
• Recent evidence of their health risks warrants serious reconsideration of their applications.
• Evidence for systemic and local infections of probiotics is discussed.
• The health risk of transmitting antimicrobial resistance by probiotics is highlighted.
• The safety of probiotics merits further assessment and investigation.

## 1 Introduction

Probiotics are live microorganisms capable of conferring a myriad of health benefits. These health claims range from preventing cardiovascular diseases and diabetes through the lowering of cholesterol and affecting the glucose and lipid levels in blood [[1], [2], [3], [4], [5], [6]], to preventing infections in the gastrointestinal tract and oral cavity [[7], [8], [9], [10]], and diarrhea caused by antibiotics [11,12]. Probiotics may also enhance the immune system [[13], [14], [15]], and cognitive function [16,17]. Probiotics exert these health effects by facilitating nutrients absorption and altering the bacterial population and dynamics in the gut [[18], [19], [20]]. To meet the demands of increasingly health-conscious and informed consumers, probiotics have now been included in various foods and health products [21,22]. They constitute a rapidly growing segment of the functional food category that promises pharmaceutical and food companies lucrative economic gains [[23], [24], [25], [26]]. Thus, it is no surprise that new and existing probiotic strains isolated from various sources such as human feces, animals, fruits, and traditional and naturally fermented foods, are being increasingly identified and characterized using classical in vitro biochemical experiments and in vivo models, as well as modern sequencing and in silico approaches [[27], [28], [29], [30], [31]].

Recent evidence has demonstrated the health risks of probiotics, but the current literature dominated by articles on the health benefits of probiotics, may mask the significance of the adverse effects. This could hamper research that contributes to addressing these health risks and concomitantly also efforts to develop safer probiotics. This review aims to highlight studies that describe how probiotics can cause opportunistic systemic and local infections especially in individuals with existing conditions, detrimental immunological effects, metabolic disturbance, allergic reactions, and facilitating the spread of antimicrobial resistance [[32], [33], [34], [35], [36]]. To offer a recent account of the literature, original research within the last five years were prioritized and discussed according to the nature of the studies in the following order of significance: clinical studies or human samples, in vivo or animal models, in situ, in vitro and/or in silico.

## 2 Opportunistic infections

**Fig. 1:**
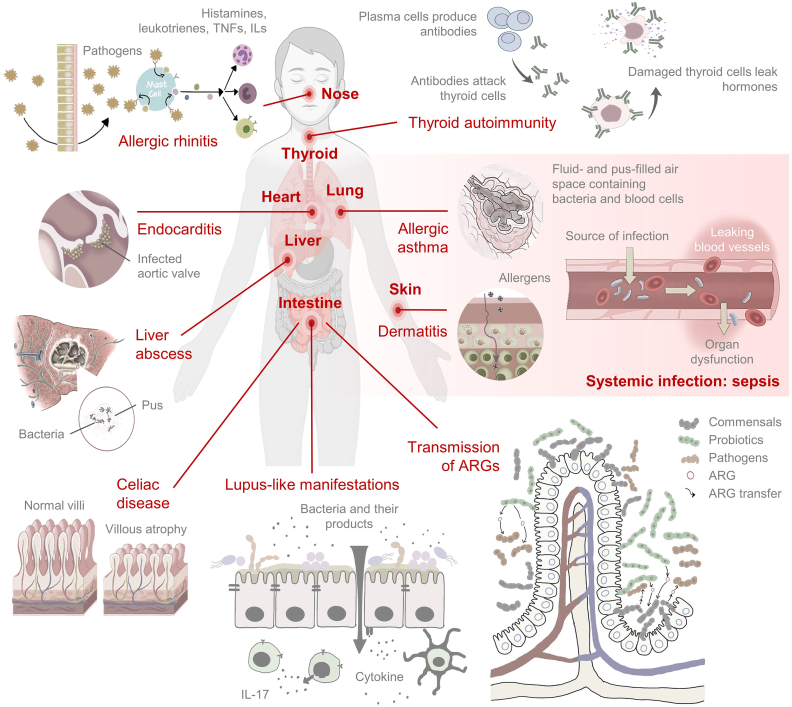
**An illustration summarizing the adverse effects of probiotics.** This figure was created with BioRender.

Previous studies have shown that probiotics can prevent infections in the respiratory and genitourinary systems [37,38]. Yet, probiotics themselves may also be the causative of infections as recent evidence has linked specific probiotic strains to sepsis, bacteremia, and fungemia, endocarditis, as well as other localized and opportunistic infections (Fig. 1).

### 2.1 Systemic infection: sepsis

When an infection triggers the overreaction of the immune system, various proteins and chemicals are released and mobilized into the bloodstream (Fig. 1). If this response becomes uncontrollable, sepsis occurs leading to extensive inflammation that may damage the tissues and organs of the host. Most sepsis-causing infections are either fungal (fungemia) or bacterial (bacteremia) in nature.

#### 2.1.1 Fungemia

In recent years, several reports have shown that probiotics may cause fungemia, many of which are reported in infants [[39], [40], [41]]. For instance, *Lactobacillus rhamnosus* GG was identified as the cause of probiotic-related sepsis in preterm infants due to its biofilm-forming capability while in full term infants suffering from immunodeficiency, treatment with the probiotic strain *Saccharomyces boulardii* may be the cause of *Saccharomyces cerevisiae* fungemia [42]. Similar cases in adults have also been reported, most of which are *Saccharomyces cerevisiae* related fungemia caused by probiotic supplementation or treatments containing *S. boulardii*. These treatments include antibiotic-associated pseudomembranous colitis, antibiotic associated diarrhea, and acute cholangitis [43]. Notably, patients subjected to the probiotic treatments all have underlying risk factors including but not limited to advanced age, carcinomas, or chemotherapy treatments, critical illness, intensive care unit (ICU) admission, central venous catheters or intravenous drug abuse, commercial probiotic use, and/or immunosuppression [[43], [44], [45]]. Consistently, one recent study conducted in Finland on human samples collected from five university hospitals over a nine-year period between 2009 and 2018, found that at least 43% of patients who had *Saccharomyces* fungemia, were subjected to treatments with *S. boulardii*, thus reaffirming the health risk of probiotic treatments not just in patients who are critically ill, but also in those with compromised gastrointestinal tract integrity [46].

#### 2.1.2 Bacteremia

In addition to fungemia, probiotics may also induce bacteremia, especially in susceptible individuals. For example, a patient with Crohn's disease and HIV, developed *Lactobacillus* bacteremia that was linked to the consumption of self-made yogurt while a 46-year-old woman with diabetes mellitus was diagnosed with *L. acidophilus* bacteremia [47,48]. Since immunosuppressive individuals are more prone to probiotic induced bacteremia, a recent study has proposed that *Lactobacillus* bacteremia could be used as an indicator for the health state of a person. The authors advocate for a full-scale investigation of the source of infection or the current immunosuppressive state when individuals with seemingly no known medical history are diagnosed with *Lactobacillus* bacteremia to reduce mortality rates associated with diseases such as diabetes and/or bacteremia itself [49]. Considering the lack of direct evidence linking administered probiotics and blood isolates, one in silico study on ICU patients reported a significantly higher risk of *Lactobacillus rhamnosus* bacteremia for probiotic treated individuals compared to untreated ones [50]. In this study, whole-genome sequencing was performed on *L. rhamnosus* isolated from blood and probiotic capsules. The results showed that all *L. rhamnosus* isolates from the probiotic capsules and more than half of the *L. rhamnosus* isolates from the blood samples of patients receiving probiotic treatment, shared the same closest reference genome, thus indicating a high correlation between the two groups of isolates. However, blood isolates obtained from patients who did not receive the probiotics showed similarity to other strains, implying that they were not derived from the probiotic product. Since lactobacilli isolated from blood of probiotic treated patients were phylogenetically inseparable from that of the administered probiotics, it infers a direct clonal transmission of probiotics [50].

*Bifidobacterium* is another common probiotic which has been reported to cause bacteremia under certain circumstances. A case report showed that a child with severe heart failure was diagnosed with *Bifidobacterium* spp. Bacteremia when receiving probiotic treatment for antibiotic associated diarrhea [51]. Moreover, one study on infants in neonatal ICU reported cases of *Bifidobacterium breve* bacteremia which were caused by probiotic administration [52]. In that study, 298 patients who were admitted to the neonatal ICU were included and given *B. breve* Yakult Strain in the period between 2014 and 2019, and the incidence rate of *B. breve* bacteremia was found to be 2% (6/298). The main trigger of bacteremia was determined to be enterocolitis and gastrointestinal perforations induced by proteins from external food sources. This finding contributes to the bacteriologic and clinical characteristics of *B. breve* bacteremia that was previously unknown [52]. Moreover, probiotic bacteremia could also significantly affect clinical outcomes of premature neonates including removal of central lines, prolonged exposure to antibiotics, and additional laboratory testing all of which, were summarized in a recent review [53].

### 2.2 Localized infection

Unlike systemic infection, a localized infection only affects an organ or one part of the body, typically resulting in localized symptoms such as swelling, pain, redness, and problems with organ function (Fig. 1). Several studies have linked probiotic consumption or administration to localized infections in different body parts including endocarditis and abscess.

#### 2.2.1 Endocarditis

Endocarditis is a fatal infection of the endocardium which is the inner lining of the heart. It is commonly caused by bacteria which have entered the bloodstream and traveled to the heart (Fig. 1). The heart can usually overcome the infection, but bacteria may be able to bypass the immune system in individuals with heart defects [54]. Molecular studies reveal that lactobacillus species enable the synthesis and lysis of fibrin clots and the breakdown of glycoproteins, which may contribute to the survival of bacteria [55]. Numerous studies have associated probiotic supplementation with the onset of infective endocarditis. For instance, *L. rhamnosus* has been determined to cause infective endocarditis in individuals with hereditary hemorrhagic telangiectasia, and those with past medical history of bicuspid aortic valve and uncontrolled diabetes mellitus [[56], [57], [58]]. While probiotic bacteremia in patients without pre-disposing factors is rare; however, one study has reported *L. rhamnosus* endocarditis in a healthy patient albeit with gingival laceration and a history of probiotic use [55]. Apart from having a history of iron deficiency anemia, the patient was determined to be healthy with no other high-risk factors. The patient had a ten-year daily probiotic intake and probiotic strains isolated from the patient did not have distinguishable differences from *L. rhamnosus*, thus indicating that long-term probiotic consumption may have contributed to endocarditis [55]. *Lactobacillus* probiotic strains are common opportunistic pathogens of infective endocarditis, especially for susceptible individuals with predisposing risk factors as systematic reviews of clinical case reports of infective endocarditis gathered between 2018 and 2020 showed that the most prevalent bacterial culture among the adult cohort was *L. rhamnosus* (80%) [59]. Moreover, one recent study conducted on animals, showed for the first time the occurrence of infective endocarditis in dogs which was associated with *Bacillus amyloliquefaciens*, a probiotic that is commonly considered beneficial to both humans and animals [60].

#### 2.2.2 Abscess

One of the most common localized infections induced by probiotics is abscess (Fig. 1). In addition to causing infections in immunosuppressed individuals, *Lactobacillus* probiotic strains such as *L. paracasei* can also act as opportunistic pathogens causing serious abscess in liver, and in the intra-abdominal and retroperitoneal parts [61]. This is in addition to masticator abscess which is caused by *Lactobacillus* infection in diabetic patients after the extraction of the wisdom tooth [62]. Clinical case studies of liver abscess have also ascertained that *L. gasseri* is more likely to cause pyogenic liver abscess in diabetic individuals and in those who have undergone surgeries [[63], [64], [65]]. The first reported case of probiotic consumption as the possible cause of a liver abscess was a 65-year-old diabetic patient with liver abscess and bacteremia resulting from *Lactobacillus paracasei* [63]. Another case report identified the occurrence of *Lactobacillus gasseri* induced bacteremia in a 59-year-old man who had a history of diabetes mellitus and several abdominal surgeries [65].

## 3 Detrimental immunological effects

Autoimmune diseases can be triggered by various causes that could be genetic and/or environmental in nature, and changes in the intestinal microbiota and the imbalance of mucosal immune response have been attributed to the pathogenesis of inflammatory and autoimmune diseases [66] (Fig. 1). Currently, one of the most promising areas of research is the therapeutic potential of probiotics in autoimmune disorders although probiotic supplementation has thus far not been conclusively shown to effectively prevent or treat autoimmune diseases or lower the levels of cytokines. Recent studies have even linked probiotics induced production of cytokines such as interleukins IL-1 β, IL-6, interferons IFN and tumor necrosis factor TNF-α, to excessive immunological effects resulting in inflammation or autoimmune disorders [[67], [68], [69], [70]]. As such, a reevaluation of probiotics applications in autoimmune diseases especially in high-risk individuals is necessary [33].

### 3.1 Clinical studies linked probiotics to elevated risk of autoimmune disorders

Several clinical trials showed that probiotic administration is ineffective for the treatment of immune-related diseases. For instance, a randomized double-blind study that examined the effects of single- and mixed-strain probiotics in severely burned patients, discovered that their IgA levels were raised by the administration of probiotics while the IL-6 levels remain unaffected [71]. As a marker for mortality in patients with septicemia, the inflammatory mediator IL-6 levels which were significantly increased in experimental and clinical studies, were unaffected by the administration of probiotics [72,73]. Likewise, a clinical trial that examined the effects of probiotic vaccination on the immune response and the incidence of influenza-like diseases in elderly people, did not show any changes in the level of influenza antibody. The relative risk of the influenza-like diseases in vaccinated, non-vaccinated, and probiotic groups was also similar [74]. Crucially, results from other clinical studies indicated that probiotics consumption could even raise the chance of developing autoimmune disorders. For instance, a 15-year survey with scheduled visits of 6520 genetically susceptible children in 6 clinical research centers worldwide found that the risk of infant suffering from celiac disease, a chronic autoimmune condition affecting the small intestine [75], increased somewhat with the introduction of probiotic dietary supplements in the first few weeks of life [76]. Another double-blind study of 96 children aged 8–17 years with newly diagnosed type 1 diabetes (T1D) was conducted to determine the effects of *Lactobacillus rhamnosus* GG and *Bifidobacterium lactis* Bb12 on beta-cell function [70]. Specifically, 96 children were randomized (probiotics, n = 48; placebo n = 48) and they were given *L. rhamnosus* GG and *B. lactis* Bb12 at a dose of 10^9^ CFU or a placebo via oral administration for 6 months, and the results of 12-month follow-up showed that there were significantly more people with thyroid autoimmunity in the probiotic group compared to the placebo group (15/46 vs. 6/46, respectively, RR 2.5, 95% CI 1.06 to 5.87; p = 0.047) [70].

### 3.2 Probiotics administration raised autoimmune markers in animals

Meanwhile, in vivo studies such as in one recent report on mice, showed that probiotic *L. reuteri* may contribute to lupus-like manifestations [77]. Specifically, the authors showed that the fecal and ileal microbiomes of lupus prone toll-like receptor 7 (TLR7) transgenic mice were enriched with *L. reuteri* while cecal metastasis study and treatment with the immunomodulatory and antitumorigenic agent imiquimod, also revealed aggravation of systemic autoimmunity in gut microbiota [77]. Moreover, *L. reuteri* could be recovered from mesenteric lymph nodes, liver and spleen of these animals, and the translocation of bacteria has been mechanistically linked to TLR7. As autoimmunity-promoting effects of *L. reuteri* have developed before detectable translocation, this suggests that metabolites secreted by *L. reuteri* could have contributed to the lupus-like manifestations [78]. Some *Enterococci* probiotic strains may also act as opportunistic pathogens. One recent study found that the translocation of *Enterococcus gallinarum* can induce autoantigens, ERV proteins, cytokines (type I IFN and other proinflammatory cytokines) and other autoimmune promoting factors (ERV gp70 and β2GPI) by affecting the differentiation of T helper cells or acting directly on colonized tissues such as liver, thus triggering autoimmune response [79]. By comparing the RNA expression profiling of *E. gallinarum*–monocolonized C57BL/6 mice with those from *Enterococcus faecalis*- and *Bacteroides thetaiotaomicron*-monocolonized mice, scientists noticed that the presence of *E. gallinarum* down-regulated ileal molecules related to barrier function (e.g., occludin, claudins, Plvap, Axin2), the mucus layer (e.g., Mucin-2), and antimicrobial defense (e.g., Reg3b, Defa2) and up-regulated those related to inflammation (e.g., Cxcr2, AhR/Cyp1a1, Enpp3) [79]. Enpp3 in particular, has been shown to increase the number of plasmacytoid dendritic cells (pDCs), which are essential for the IFN signature in human systemic lupus erythematosus [80,81]. Based on previous research showing that endogenous retrovirus glycoprotein 70 (ERV gp70) drives lupus kidney disease via TLR7 and these results [82], scientists hypothesized that the liver-resident *E. gallinarum* may induce hepatic overexpression of ERV gp70 that fuels the formation of *anti*-ERV immune complexes and systemic autoimmunity since they discovered that (NZW × BXSB)F1-derived hepatocytes cocultured with *E. gallinarum* isolated from an (NZW × BXSB)F1 liver, induced numerous autoimmune-promoting factors like the autoantigens ERV gp70 and β2GPI [79]. Another recent study that examined whether oral administration of *L. plantarum* CJLP243, CJW55-10 and CJLP475 could induce cell-mediated immunity in immunodeficient mice, showed that the consumption of all three probiotic strains promoted interferon-γ (IFN- γ), IL-1 β, IL-6, IL-12, and TNF- α [83]. In addition, inducible nitric oxide synthase levels and costimulatory molecules (CD80 and CD86) were also up regulated in BMDMs after treatment with these probiotics, while the cytotoxicity of NK cells and the proliferation of immune cells were also elevated [83]. In agreement with the clinical case studies and in vivo data, recent systematic review and meta-analysis also concluded that probiotic supplementation had either no effect on the disease or negatively affect the health of the host through elevation of cytokines such as IL-6, especially in those with existing conditions such as rheumatoid arthritis and chronic kidney disease [84,85].

## 4 Allergic reactions

Allergic diseases including skin allergies, allergic asthma and rhinitis, and food allergies have become increasingly common (Fig. 1). Alternative treatments such as probiotic therapy have been explored and attempted for the treatment of allergic disorders but with inconclusive or contradictory results. In vitro and in vivo studies have shown the prospect of probiotics in treating allergies, but clinical evidence is still lacking due to significant heterogeneity, including different lifestyles, clinical phenotypes, airway microbiomes, health conditions, gender, and age, among individuals [[86], [87], [88], [89]]. Moreover, probiotic treatments may lead to increased sensitization in high-risk adults and children [90,91]. In newborn microbiota for instance, allergic disorders are normally preceded by decreased in microbial diversity including fewer lactobacilli and bifidobacteria [92], and perinatal use of probiotics has been associated with more frequent IgE sensitization to the dander of cats and dogs, during later developmental stages e.g., at 13 years of age [90].

### 4.1 Skin allergy

Atopic dermatitis (AD) is a chronic, inflamed skin condition that affects 10–20% of people, particularly babies [93] (Fig. 1). Previous studies have indicated that probiotics can prevent and treat AD in both children and adults [94,95] but the implementation of probiotics in the management of AD still lacks conclusive evidence as the results of some studies in the current literature were positive while others reported no effect. Moreover, the same probiotic strain that proved effective against AD in one study group may not be effective in another [93,96]. For instance, infants aged 1–36 months with moderate or severe atopic dermatitis were given synbiotic or placebo, and then their severity of atopic dermatitis (SCORAD) was scored, revealing that there was no significant difference in the mean decrease of total SCORAD between placebo (22.3) and synbiotic groups (24.2) [97]. The results of two other studies on the on the impact of *Lactobacillus rhamnosus* on infantile atopic dermatitis revealed that there was no significant difference between the placebo group and the control group at the conclusion of probiotic treatment for clinical symptoms (SCORAD, pruritus, sleep loss), or immunological parameters [98,99]. Additionally, the first six months of life were spent administering either *Lactobacillus acidophilus* (LAVRI-A1) or a placebo to newborns of allergic women (n = 231). At six and twelve months, the infants were evaluated for AD and other symptoms, and it was discovered that the probiotic group's sensitization rate was significantly higher than that of the placebo group [91]. This inconclusive and sometimes contradictory data was well-reviewed by Tan-Lim et al. (2021) [88]. Importantly, the authors highlighted the occurrence of adverse events through a comprehensive systemic and meta-analysis of probiotic use in pediatric AD where the use of mixed-group probiotics for instance, may cause more adverse events compared with placebo based on low-quality evidence (RR = 1.06, 95% CI 0.02–51.88) [88]. To complicate matters, variations in strain specificity, time, and administration time could also affect clinical outcomes. These factors should therefore be considered during prescription [100]. Meanwhile, clinical studies on other skin allergy diseases such as eczema and atopic sensitization show that probiotics were ineffective in preventing or reducing the prevalence of the respective disease conditions [101].

### 4.2 Allergic rhinitis and asthma

In addition to skin allergies, the use of probiotics in the prevention or treatment of allergic rhinitis and asthma is also a cause for concern (Fig. 1). While animal studies under well-controlled conditions have provided substantial evidence that certain probiotic strains may help prevent wheezing and asthma, however, results from clinical studies in children and infants, including prenatal and postnatal administrations, were less encouraging [102]. The current literature shares the opinion that there is little evidence to recommend the use of prebiotics or synbiotics to prevent childhood asthma and allergic rhinitis [102,103]. Furthermore, recent studies have showed that the consumption of probiotic supplement is not associated with a lower risk of asthma in infants [[102], [103], [104]], and that some probiotics could even be harmful if used incorrectly [105]. For instance, the prevalence of allergic rhino-conjunctivitis increased in patients taking probiotics during perinatal and childhood [105]. Additionally, the complex interactions between the host and the environment have also caused significant variations in the effects of probiotic while the response of gut microbiota of different host to the probiotics, may lead to markedly varied effects including adverse ones [86,96], hence the need for more conclusive clinical evidence before probiotics can be safely prescribed for patients with asthma or allergic rhinitis.

### 4.3 Food allergy

Studies on food allergy have generally focused on infant populations, particularly the relationship between breastfeeding and infant development, as breastfeeding is the most important postnatal factor that supports microbial colonization in gut of infants and drives the development of the neonatal immune system [106]. Poorly established gut microbiota in early infancy has been identified as the key factor for the development of food allergy [107], thus it is no surprise that infant milk formulations have been fortified with probiotics. The effects of early exposure to milk on the increase in allergic diseases (mainly food allergies and atopic dermatitis) are still controversial. Some experimental results suggest that early exposure to CMP as a supplement to breastfeeding may promote tolerance and is associated with a reduced risk of milk allergy [108,109], while a recent review by searching literatures have found that early-life milk supplementation may even accelerate the onset of IgE sensitization and food allergies [110]. These conflicting results may be due to confounders like family history of atopy, the number of outcomes, the length of breastfeeding, weaning, age at analysis, definition [87,110]. Recent studies have shown that the intake of probiotics can reduce the risk of food allergy in children [[111], [112], [113]]. Although some promising results are mainly related to the effects of specific probiotics on intestinal microbiota, the clinical evidence for the beneficial role of probiotics in CMA is still inconclusive [114,115]. A recent systematic review considered a randomized trial involving 895 pediatric patients with CMA showed that probiotics relieved the symptoms, but the results of SCORAD were not accurate and no definite conclusion could be drawn [116]. Much like skin allergies, studies on the effect of probiotics in cow's milk allergy, were inconsistent due to the scarcity and heterogeneity in terms of the health conditions or family hereditary history of studies in the current literature thus, hampering effective management and regulation of probiotics use in breastfeeding mothers and infants [87].

## 5 Metabolic disturbance

Obesity, which is becoming more prevalent globally, has been associated with the development of metabolic syndrome [117]; a disorder that is characterized by dyslipidemia, dysregulated glucose homeostasis, deteriorated liver and kidney function, elevated arterial blood pressure, as well as excess body weight, abdominal obesity, and/or insulin resistance [118]. Metabolic syndrome typically increases the risk of cardiovascular disease and type 2 diabetes [119]. Numerous studies have shown that changes in the composition of the gastrointestinal microbiota play a role in the development of obesity-related insulin resistance [[120], [121], [122]], which then causes metabolic disorders. To overcome the latter, probiotics have been used but the results, whether in prevention or controlling of the disorders, were not overwhelmingly positive while several studies have even reported unexpected adverse effects.

### 5.1 Probiotics are linked to disorders associated with defective lipid metabolism

Several in vivo studies showed that probiotics were not only ineffective in regulating lipid metabolism but even triggered adverse responses in mice. For instance, a recent in vivo study of rats has revealed that the gut microbiome-derived lactate promotes anxiety-like behaviors through G protein-coupled receptor 81 (GPR81)-mediated lipid metabolism pathway [123]. Rats were randomly assigned to either a control group or an anxiety group and subjected to various stressful situations and a series of behavioral tests for 30 days. The fecal samples were then processed for 16S rRNA sequence, untargeted metabolomic, histological, PCR, and Western blot analyses. The findings demonstrated that anxious rats had significantly higher levels of lactic acid, and that lactic acid produced by intestinal microorganisms activated GPR81 lactate receptors, causing anxiety-like behaviors like psychomotor discomfort and learning and memory impairment by regulating lipid metabolism disorders brought on by fat decomposition. Additionally, the activation of GPR81 in the liver of these rats resulted in an inhibition of the adenylate cyclase (AC)-protein kinase A (PKA) pathway of lipolysis and an increase in tumor necrosis factor (TNF), which causes inflammation [123]. Other recent studies have also shown that *Lactobacillus* causes a large accumulation of bacteria in the small intestine and that the lactate produced by the lactobacillus-fermented foods can further contribute to brain fogginess [124], memory and cognitive impairment [125]. Moreover, another study found that *E. faecalis* probiotic strain causes hypertension and renal damage in rats by disrupting lipid metabolism [126]. The use of probiotic also increased the level of HDL-C, whose extremely high or low levels were associated with an increased risk of death [127,128]. Meanwhile, one study reported no significant changes in the diet, body weight, or serum triglyceride levels of mice due to probiotics administration [129]. Consistently, in silico studies also showed that the effect of probiotics on metabolic syndrome was clinically insignificant [130]. Moreover, although probiotics have been found to change the microbiota to enhance metabolic indices and weight reduction, their effects on specific types of central adipose tissue, primarily viscera and subcutaneous adipose tissue, were inconsistent [131].

### 5.2 Probiotics appear ineffective in treating insulin resistance

Insulin resistance is one of the leading causes of gestational diabetes mellitus, a type of glucose intolerance that occurs during pregnancy's second and third trimesters [132]. Insulin resistance can be exacerbated by risk factors such as obesity, pregnancy, and lifestyle [133]. Current literature suggests a possible treatment of probiotics on glucose metabolic disorders by significantly reducing the fasting plasma glucose (FPG) and glycated hemoglobin (HbA1C) [134,135], especially in obese pregnant women where the intestinal flora disorders could lead to metabolic disorders and gestational diabetes mellitus [101,136,137]. However, several recent clinical studies have shown that probiotics consumption only shows limited relief for diabetes, especially gestational diabetes [138], or had no effect in reducing the risk of gestational diabetes mellitus or improving glucose metabolism in overweight and obese women no matter the use of probiotics either as a single drug or in combination with prebiotics [139]. Another study that examined the effects of multi-species synbiotics on glucose metabolism, intestinal microbiota, intestinal permeability, neutrophil function, and quality of life in diabetic patients, also showed that glucose metabolism as the primary outcome, was unchanged during the intervention with a multispecies synbiotic in patients with diabesity [140]. Even worse, a silico study as to the effect of probiotics on the prediction of GDM demonstrated that compared with placebo group, probiotics increase the risk of pre‐eclampsia compared to placebo (RR 1.85, 95% CI 1.04 to 3.29; 4 studies, 955 women; high‐certainty evidence) and may also increase the risk of hypertensive disorders of pregnancy (RR 1.39, 95% CI 0.96 to 2.01, 4 studies, 955 women) [141]. In non-diabetic patients, including obesity [142], depression [143], polycystic ovary syndrome (PCOS) [144], probiotics did not play an effective role in regulating sugar metabolism such as reducing fasting plasma glucose and quantitative insulin sensitivity check index or influencing pancreatic β-cell function. Although a study of the effects of probiotic supplementation on exercise and metabolic parameters in patients with Parkinson's disease found that probiotic supplementation significantly reduced insulin levels and increased insulin sensitivity to improve cognitive impairment compared with placebo group, probiotic intake had no significant effect on other metabolic characteristics such as total cholesterol and MDA [145]. Therefore, larger clinical trials are needed to verify the effects of probiotics on metabolites.

## 6 Facilitating the trafficking of antimicrobial resistant genes (ARGs)

Probiotics are known to harbor resistant genes in the form of mobile genetic elements such as plasmids and transposons [[146], [147], [148]], and the risk of transferring ARGs to host microbiota leading to the establishment of ARG reservoirs in natural environments, endanger human health especially when the ARGs are acquired by pathogens which would limit the options of effective antibiotics for treatments (Fig. 1). This is by far the most discerning concern of probiotics as the literature is overwhelmed by numerous reports and reviews on this topic [24,[147], [148], [149], [150], [151], [152], [153], [154], [155], [156], [157]]. While a large body of evidence has reported multidrug resistant probiotics and the presence of ARGs in probiotics from human samples, animals, foods, and health supplements [[158], [159], [160], [161], [162], [163], [164], [165]], however, direct evidence that demonstrate probiotic-to-pathogen transfer of ARGs in human samples or clinical studies, and from animal models, is scarce even though such evidence has been reported since 2007. In that first study conducted on gnotobiotic rats, *Lactobacillus plantarum* isolated from fermented dry sausages was able to transfer their plasmids harboring tetracycline and erythromycin resistance genes *tet(M)* and *erm(B)* to *Enterococcus faecalis* JH2-2 [166] while in 2008, the first transfer of *vanA* which is responsible for vancomycin resistance from enterococci to a commercial *Lactobacillus acidophilus* strain, was demonstrated not just in vitro but also in vivo and at high frequencies when transiting through the digestive tract of mouse even without antibiotic pressure [167]. Consistently, a higher transfer frequency of an *erm(B)* containing plasmid pLFE1 from *L. plantarum* to *E. faecalis*, was observed in gnotobiotic rats compared to in vitro filter mating experiments in the absence of antibiotics. When erythromycin was administered, the transfer rate increased significantly to almost 100% [168].

### 6.1 Evidence for the expansion of gut resistome in humans

Yet, to-date, only one study published recently in 2021, has to the best of our knowledge, examined the effect of probiotics on the ARG reservoir in humans [169]. Metagenome analyses revealed that although supplementation of commercially available probiotics resulted in a reduction of ARGs in the gut of healthy antibiotics-naïve and colonization-permissive individuals, but, when administered with antibiotics, probiotics resulted in an expansion of the resistome in the lower gastrointestinal tract in humans and mice. Interestingly, the probiotics-associated expansion of resistome in the mucosa of the gastrointestinal tract was achieved through the increase in bacteria carrying vancomycin resistance genes and not the resistance genes from probiotics. Notably, these effects were absent in stool samples which highlighted the significance of analyzing the metagenomes of gut resistome through direct sampling [169]. Previously, another study examined the effects of more than 1000 non-antibiotic drugs on human gut microbiota and found that those with anti-commensal activities also exert antibiotic-like side effects. Importantly, these drugs could evoke the same resistance mechanisms as that induced by antibiotics since there is a correlation between the susceptibility profiles of bacterial species to antibiotics and to the non-antibiotic drugs, thus highlighting the risk of using alternative antimicrobial agents including probiotics as biotherapeutics [170].

### 6.2 In vivo and in situ evidence for the transmission of ARGs

In vivo and in situ studies such as in one recent report on fermented soybean meal and in the digestive tract of pigs, showed intergenic transfer of plasmids harboring the vancomycin (*vanA*) and chloramphenicol resistant genes between probiotic enterococci as confirmed by multilocus sequence typing (MLST) and pulsed field gel electrophoresis (PFGE) analysis of the transconjugants [171]. Another study conducted on rats also reported conjugal transfer of erythromycin and tetracycline resistant genes *erm(B)*, and *tet(M)*, *tet(L)* and *tet(W)* genes, from *L. salivarius* or *L. reuteri* to *E. faecalis* JH2-2. Importantly, when examined in situ during food fermentation of chicken sausage, fermented milk or idli batter, pathogens *Listeria monocytogenes* and *Yersinia enterocolitica* introduced deliberately to represent contaminants, were found to be resistant to erythromycin and tetracycline [172]. A study that compares the resistance profiles of lactic acid bacteria from conventional poultry chicken and organic chicken, showed that there were about 5-7 orders of magnitude higher erythromycin, tetracycline, and vancomycin resistant bacteria in conventional poultry chicken [173]. These bacteria were identified through repetitive-PCR profiling and 16S rRNA gene sequencing as *Enterococcus faecium*, *Enterococcus durans*, *Lactobacillus plantarum*, *Lactobacillus pentosus* and *Lactobacillus salivarius*, and they harbor transposons-associated resistant genes *erm(B)*, *msr(C)*, *msr(A/B)*, *tet(M)*, *tet(L)* and *tet(K)*. Further conjugative experiments conducted in vitro and in rats showed that *E. faecium* M3G and *L. plantarum* S11T could transfer their erythromycin and tetracycline resistant genes to *E. faecalis* JH2-2 [174]. A more recent study that examined lactic acid bacteria in starter and protective cultures were phenotypically resistant to tetracycline, kanamycin, and chloramphenicol with *aph(3’)-IIIa* and *cat* being the most prevalent resistance genes identified [174]. Importantly, both in vitro filter mating and conjugal experiments conducted in situ on food matrix showed that tetracycline resistant genes *tet(K)* and *tet(M)* from *Lactococcus lactis*, *Pediococcus pentosaceus*, and *Lactobacillus plantarum* could be transferred to *E. faecalis* JH2-2 [174].

### 6.3 Indirect evidence inferring transmission of ARGs

Indirect evidence from studies conducted on commensals that carry multidrug resistant plasmids showed the feasibility of plasmid transfer to gut bacteria after colonization of the murine gut in mice. Through evolutionary adaptation to antibiotic exposure, *Escherichia coli* K-12 MG1655 carrying the RP4 plasmid that harbor the *blaTEM*, *tet(A)* and *aphA* genes responsible for resistance to ampicillin, kanamycin, and tetracycline, showed multidrug resistance phenotypes among other changes such as improved growth and biofilm formation [175]. The latter is associated with the upregulation of *tnaA* gene encoding for tryptophanase-catalyzing indole formation that could sustain higher population density in the evolved strains. Other genotypic alterations include the upregulation of chromosomal genes that encode for efflux pumps, outer-membrane protein, multidrug-resistance protein, and macrolide export proteins, as well as the downregulation of plasmid-harboring genes that encode for conjugal transfer protein, replication protein, beta-lactamase TEM precursor, aminoglycoside 3′-phosphotransferase, and tetracycline resistance protein A. Importantly, the RP4 plasmids could be transferred to other gut bacteria including *E. coli*, *E. fergusonii*, *K. pneumonia*, *K. singaporensis* and *B. fungorum*. Collectively, the adaptation induced genotypic and phenotypic modifications enabled prolonged survival time of plasmid-carrying strains which in turns, facilitated the transfer of plasmids in the murine gut of mice [175].

Employing a simulated colon set up called the mucosal simulator of the human intestinal microbial ecosystem (M-SHIME), one study showed using the same commensal *E. coli* model MG1655 that the p5876 plasmid originating from broiler chicken and carrying genes responsible for cefotaxime, tetracycline, and sulfamethoxazole resistance, can be transferred to indigenous coliforms in the lumen and mucosa [176]. This transfer is not at all affected by the meal size and digestion. Significantly, 96% of resistant colonies contained the p5876 plasmid as determined by PCR which the authors attributed to the direct acquisition of plasmid from the MB6212 strain, indirectly through another transconjugant, or through vertical transfer during growth [176]. In another study, the plasmid pSELNU carrying the *lnuA* gene responsible for lincomycin resistance was shown to transfer from *Staphylococcus equorum* KS1030 to *Staphylococcus saprophyticus* KM1053 even in the absence of antibiotic pressure when transiting through the murine intestine of mice [177]. In contrast, when examined in situ on soybean matrix, pSELNU plasmid which is commonly found in *Staphylococcus equorum* strains in high-salt fermented food, can only be transferred to *Staphylococcus saprophyticus* KM1053 in the presence of lincomycin, thus implying a higher efficiency of horizontal gene transfer under in vivo conditions [177].

### 6.4 Metagenomics studies implicating transmission of ARGs along the food chain

In humans, a metagenomics study of 162 individuals composing of 38 Chinese, 85 Danish and 39 Spanish, identified a total of 1093 unique ARGs from 4.1 million gut genes or 0.266% of the gut microbiome which the authors found to be much higher than that in other natural environments such as soil and water [178]. The ARGs were subsequently grouped into 149 different resistance gene types of which 95 and 54 were determined to be single- and multi-drug resistance gene types. Mapping the gene types to the individuals in the different populations revealed 133 gene types among the Chinese and Danish populations while the Spanish population harbored 128 gene types. When examining the relative enrichment of genes based on sequencing coverage, the authors found that the proportion of ARGs to the total number of genes in gut was highest in Chinese individuals (0.94%), followed by Danish individuals (0.89%) and Spanish individuals (0.44%). Notably, one Chinese sample had 89 resistance gene types which is the highest in the pool while the lowest belongs to one Danish sample with 33 gene types. The ARGs *ant6ia*, *bacA*, *vanRA*, *vanRG*, *tet(32)*, *tet(40)*, *tet(O)*, *tet(Q)* and *tet(W)*, were identified in all samples from the three populations while *erm(B)* was found in all but one sample. Interestingly, a high abundance of genes conferring resistance to tetracycline which is commonly used in animal feeds, including *tet(36)* that was first identified from a *Bacteroides* strain in swine manure pits in the US, were found in nearly 16% of Chinese individuals but absent in the other cohorts [178]. Critically, a follow up study from the same group found that antibiotics used in animal husbandry have greater impact in enriching ARGs in the human gut than that of antibiotics used in human medicine [179]. This is consistent with previous studies that also revealed a common pool of ARGs in humans and in the environment including animals and soil. While indirect, these studies infer that ARGs are disseminated and accumulated along the components of the food chain, beginning from farm to fork [[180], [181], [182]]. Thus, it is conceivable that probiotics which are widely applied in starter cultures, fermented foods and beverages, and health supplements, may facilitate or exacerbate the spread of ARGs.

### 6.5 In vitro evidence of ARG transfer

In recent years, several in vitro evidence have demonstrated the feasibility of ARG transfer from probiotic strains to representative pathogens. One such example is the transfer of the tetracycline resistant gene *tet(K)* by electroporation and transformation, from *Lactobacillus fermentum* to *Citrobacter freundii*, a Gram-negative gut commensal which was determined to have no tetracycline resistant genes nor is it phenotypically resistant to tetracycline examined [183]. Another study showed that Macrolide-Lincosamide-Streptogramin (MLS) resistance from *Lactobacillus fermentum*, *Enterococcus hairae* and *Enterococcus faecalis*, isolated from animals and food such as idli batter, chicken and sheep intestine, can be transferred to *E. faecalis* JH2-2, *Lactococcus lactis* and *Acinetobacter* examined through filter-mating studies [184]. The lactic acid bacteria contained *erm(B)*, *mefA/E* genes *msrA/B* and notably, the transconjugants tolerated 3–4 folds higher amounts of erythromycin and clindamycin [184]. Another study on bifidobacteria showed through conjugation assays that the erythromycin resistant gene *erm(X)* can be transferred from *Bifidobacterium catenulatum* subsp. *Kashiwanohense* DSM 21854 to other bifidobacterial strains including *Bifidobacterium longum* subsp. *Suis* DSM 20211 [185]. Further whole-genome sequencing and comparative genomic analysis revealed that the *erm(X)* gene is located on the genomic island BKGI1 which is highly unstable and excisable in some bifidobacterial strains, thus is conjugally mobile and transferable. Since BKGI1 homologs are also present in other bifidobacterial strains especially *B. longum*, the genomic island BKGI1 therefore mediates the spread and integration of *erm(X)* in bifidobacteria [185]. Adaptive evolution of *Lactiplantibacillus plantarum* isolated from a commercially available probiotic health product led to a daughter strain with increased resistance to amoxicillin-clavulanic acid and clarithromycin which are used to treat *Helicobacter pylori* infections [186]. The increased resistance to clarithromycin and amoxicillin-clavulanic acid is also accompanied by the development of resistance to other classes of antibiotics such as fluoroquinolones and cephalosporins but resulted in a decrease in resistance to aminoglycosides, tetracyclines, and rifampicin. Comparative genomic analysis of the adapted antibiotic-resistant and parental strains detected point mutations and larger-scale genomic rearrangements that may account for the development of resistance such as the insertion of the transposase gene ISLpL3 into the esterase gene. Interestingly, the antibiotic-resistance strain is accompanied by an increased in virulence in *Drosophila melanogaster* which is a natural host for the *L. plantarum* symbiont, assessed through viability and reproduction studies such as egg numbers, embryonic death, and DNA damage in enterocytes of the flies [186].

### 6.6 In silico studies in support of ARG transfer

Given the availability of increasing number of complete probiotic bacteria genomes and the development of bioinformatic tools such as ARG-ANNOT and Resfinder for the detection of existing and putative ARGs, a recent in silico study has analyzed 126 complete probiotic bacterial genomes and detected many ARGs some of which, are known to be transferable [187]. The authors found that the tetracycline resistant gene *tet(W)* found in both *Bifidobacterium* and *Lactobacillus* was the most abundant [187]. Another study that analyzed 47 shotgun sequencing datasets from probiotic samples consisting of 20 singles isolates and 27 metagenomes, detected more than 70 ARGs that operate through known mechanisms such as antibiotic efflux, inactivation or reduced permeability, and antibiotic target alteration, protection, or replacement [188]. They offer tolerance to a broad range of antibiotics with the most common being rifampicin, extended-spectrum beta-lactamase (ESBL) and tetracyclines as evidenced by the highest abundance of *rpoB* mutants, *TEM-116* and *tet(W/N/W)* genes in the analysis. Additional analysis on the mobilome, plasmids and phages also concluded that many of these ARGs are transferable [188]. Another in silico combined literature analysis on bifidobacterial, revealed that resistance to aminoglycosides, polypeptides, quinolones, and mupirocin were the most abundant while resistance to erythromycin, tetracycline, fusidic acid, metronidazole, clindamycin, and trimethoprim, were variable [189]. The authors identified 3520 putative ARGs from 831 bifidobacterial genomes matching 38 unique reference ARGs through a BLASTp search excluding the *cmX* and *tet(Q)* harboring plasmids already known to be present in bifidobacteria. These ARGs confer tolerance to aminoglycosides, macrolides, tetracyclines, trimethoprim, fluoroquinolone, and polypeptides antibiotics many of which, are transferable. *tet(W)* and *erm(X)* are the most abundant ARGs and they have different distribution traits. Consistent with other studies, *rpoB* mutants were present together with another housekeeping gene, the mupirocin-resistant isoleucyl-tRNA synthetase *ileS*. Tetracycline resistant genes *tet40*, *tetC*, *tetO*, and *tet(W/N/W)* were also present. Other genes such as *cmX*, *catl* and *cat-TC* for chloramphenicol resistance; *dfrF* for trimethoprim resistance; *QnrB19* and *QnrB10*, *EfmA* and *EfrB* for fluoroquinolone resistance; *bcrA* for polypeptide resistance; and *parY* for aminocoumarin resistance, were represented in the analysis [189].

## 7 Conclusion and outlook

The positive effects of probiotics in the prevention and treatment of diseases have been well-documented but doubts on their dosage and long-term safety especially in patients with underlying health conditions, persisted. It is worth noting that many of the detrimental health effects are secondary infections that occur in immunocompromised patients and similar incidences in healthy individuals are relatively uncommon [190]. In some cases, administration of probiotics was ineffective e.g., in reducing the mortality rate and length of hospitalization [191]. A recent authoritative review using random-effect meta-analysis and trial sequential analysis concluded that probiotic administration was not only ineffective in reducing the rates of ventilator-associated pneumonia and diarrhea in critically ill patients but is also associated with a significantly higher risk of adverse effects [192]. Moreover, poorly designed clinical studies and bias result assessments, have been determined as the main reasons for the apparent inconclusiveness on the clinical use of probiotics [191]. For these reasons, medical practitioners have cautioned against the use of probiotics, especially in critically ill patients [[190], [191], [192], [193]]. On the other hand, it has been documented that the administration of heterogenous but not single-strain probiotics, could significantly reduce surgery related complications, as well as surgical and non-surgical site infections [194]. These effects are however irregular with large variation in outcomes due to the non-homogeneity in probiotic types, amount, and frequency of administration across studies [194,195]. This predicament is further compounded by inherent microbiome differences across individuals [190].

Information on the interaction of probiotics with known drugs is also scarce [196]. For instance, probiotics have been hypothesized to antagonize the action of warfarin, which is an anticoagulant [197]. In another example, the probiotic *S. boulardii*, may also interact with antifungals, thus reducing the efficacy of this probiotic [198]. Furthermore, probiotics may also affect the bioavailability, efficacy, and safety of drugs [196]. One reason for the lack of such studies is the fact that probiotics are classified as dietary supplements, nutraceuticals, or food, which are regulated much less stringently than medical and pharmaceutical products [25,199]. Thus, research that focuses on the interactions of probiotics with a particular food, nutrient, and/or clinically important drugs especially those administered to chronically ill patients, is necessary.

Emerging technologies that leverage genetic engineering and gene editing techniques have been employed to develop probiotics as live biotherapeutics [200,201]. For instance, probiotics have been engineered to secrete various compounds such as interleukins, linoleic acid, therapeutic enzymes, and antimicrobial proteins, to suppress tumor growth, inhibit pathogens, reduce inflammation, and treat metabolic disorders, or to express antigens that can elicit antibody production or to generate signals for targeted vaccination and diagnosis [[202], [203], [204], [205], [206], [207], [208], [209]]. Currently, authorities in the US and Europe require that new genes introduced are incorporated into the genome of probiotics and the engineered bacteria should be free from ARGs carried on mobile genetic elements [200]. To fully harvest the benefits of probiotics, such regulations should be strictly enforced and introduced, if not already, globally, especially in developing countries where public health entities are consistently challenged.

Removal or curing of plasmids harboring ARGs have also been successfully applied to a commercial probiotic strain *Lactobacillus reuteri* ATCC 55730, where two plasmids harboring tetracycline and lincosamide resistance genes *tet(W)* and *Inu(A)* were removed to generate *L. reuteri* DSM 17938, a new daughter strain that still retain the probiotic properties when tested in vitro and in clinical trials [210,211]. Given the increased precision and efficiency of novel methods and technologies such as the clustered regularly interspaced short palindromic repeats (CRISPR) and CRISPR associated (Cas) proteins, ARGs and virulence genes in probiotics can be targeted for removal by CRISPR-Cas systems such as the successfully elimination of tetracycline resistant gene *tet(W)* in *Bifidobacterium animalis* subsp. *lactis* [212,213]. This would enable broad application of existing and new probiotic strains especially in at-risk individuals.

In conclusion, this review serves to inform consumers, food scientists, and medical practitioners, on the health risks, while also encouraging research that will focus on and clarify the adverse effects of probiotics. This knowledge would not only benefit the use of probiotics for gut health but could also be expanded to microbiomes beyond the gut such as skin, oral cavity, respiratory tract, and lungs.

## Funding statement

Dr. Aloysius Wong was funded by Wenzhou-Kean University Student Partnering with Faculty/Staff (SpF) research program (SpF2021002).

## Data availability

No data was used for the research described in the article.

## CRediT authorship contribution statement

**Xiangyi Liu:** Writing – review & editing, Writing – original draft, Investigation, Formal analysis, Data curation, Conceptualization. **Haiyi Zhao:** Writing – review & editing, Writing – original draft, Investigation, Formal analysis, Data curation, Conceptualization. **Aloysius Wong:** Writing – review & editing, Writing – original draft, Supervision, Resources, Project administration, Methodology, Investigation, Funding acquisition, Formal analysis, Data curation, Conceptualization.

## Declaration of competing interest

The authors declare that they have no known competing financial interests or personal relationships that could have appeared to influence the work reported in this paper.
